# Cor Triatriatum Sinister in a Young Adult: An Unusual Cause of Syncope

**DOI:** 10.7759/cureus.61039

**Published:** 2024-05-25

**Authors:** Jose Alberto Domínguez-López, Luis E Mendoza-Razo

**Affiliations:** 1 Genetics, Universidad Autónoma de Chiapas, Instituto de Salud del Estado de Chiapas, Tuxtla Gutiérrez, MEX; 2 Intensive Care Unit, Instituto de Salud de Chiapas, Tuxtla Gutiérrez, MEX

**Keywords:** cor triatriatum, cardiovascular disease, genetic syndromes, adult congenital heart disease (achd), valve disease

## Abstract

A 25-year-old male with no prior medical history presented with a one-month history of nausea, weight loss, and dyspnea that progressed to syncope. The initial echocardiogram showed a dilated right ventricle with signs of systolic failure. The patient was admitted for suspected pulmonary embolism, but chest computed tomography (CT) revealed interstitial pneumonia. A transthoracic echocardiogram on day 6 of admission diagnosed cor triatriatum sinister (CTS), severe pulmonary hypertension, chronic cor pulmonale, and reduced right ventricular function. The patient was managed conservatively in the intensive care unit (ICU) without the need for mechanical ventilation and discharged after clinical improvement. This case highlights the importance of the early diagnosis of rare congenital heart defects such as cor triatriatum sinister, which can present with nonspecific symptoms and rapidly progress to right heart failure.

## Introduction

Cor triatriatum sinister (CTS) is an uncommon heart defect found in approximately 0.1%-0.4% of individuals with congenital heart conditions. In the typical form, the left atrium (LA) is divided by a fibromuscular membrane into two sections: a proximal chamber receiving all pulmonary venous blood and a distal portion containing the atrial appendage and the mitral valve vestibule, also known as the "true" atrium [1,2].

Cor triatriatum sinister is a rare congenital abnormality characterized by various clinical presentations, influenced by the extent of membrane blockage and concurrent heart issues. The scarcity of documented cases in the medical literature has led to limited data, primarily comprising small case studies involving both children and adults. The estimated prevalence in the overall population is expected to be below 0.004%. Since 1968, fewer than 350 instances have been documented. There appears to be a slight male bias in occurrence. In pediatric cases, this anomaly is linked with other congenital heart issues in around 80% of instances, commonly seen alongside conditions such as ostium secundum atrial septal defect and anomalous pulmonary vein return. The prevalence of asymptomatic cases among children with cor triatriatum sinister ranges widely (0%-75%), making it challenging to determine an exact figure. In adults, common symptoms include exertional dyspnea, orthopnea, and palpitations. Patients with obstructive membrane physiology often have associated heart defects and are prone to conditions such as congestive heart failure, pulmonary hypertension, infections, and bleeding episodes [1-3].

This condition involves a posterior-superior chamber receiving pulmonary veins and an anterior-inferior chamber connecting to the mitral valve. Various theories exist regarding the origin of cor triatriatum sinister, with the prevailing explanation attributing it to the incomplete fusion of the pulmonary vein with the left atrium during fetal development.

The natural history of cor triatriatum sinister (CTS) depends on obstructive physiology and congenital anomalies associated with it. In the obstructive form, it typically presents as heart failure and pulmonary hypertension. The severity of heart failure correlates with the degree of obstruction caused by the membrane [2-4].

## Case presentation

A previously healthy 25-year-old male with no medical history began experiencing symptoms such as nausea, weight loss, and dyspnea in January 2023 but did not seek medical attention until he experienced a one-minute syncope on February 23, 2023.

The patient began experiencing symptoms in January 2023, including nausea, weight loss of approximately 5 kg, and progressive dyspnea that eventually became present even at rest but did not seek medical attention. On February 23, 2023, the patient experienced a one-minute syncope and went to a private clinic, where he was treated with bromhexine, moxifloxacin, and beclomethasone and was advised to undergo a dobutamine stress test and echocardiogram. The first echocardiogram showed high atrial values within normal limits, a small left ventricle with paradoxical movement and an ejection fraction of 60%, and a very dilated right ventricle with signs of systolic failure.

**Figure 1:**
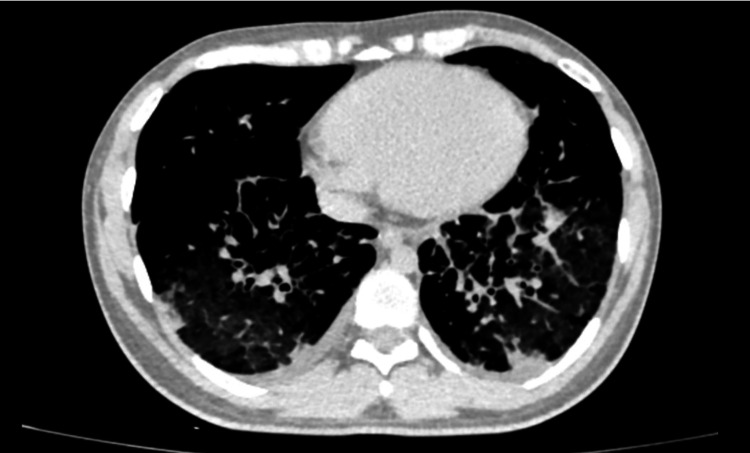
Chest computed tomography scan Consolidation in the right upper lobe with air bronchogram and posterior middle lobe with air bronchogram inside. In the left lung, the presence of consolidation with air bronchogram in the posterior region and in the posterior inferior lobe

**Figure 2:**
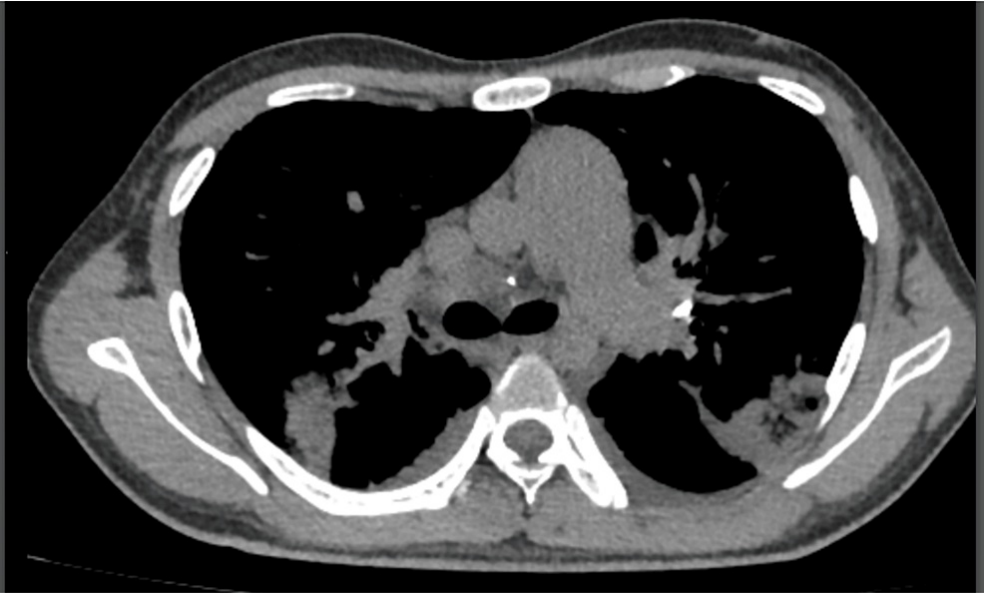
Chest computed tomography scan Posterior pulmonary atelectasis in both lungs and inferior lobes with consolidation zones

The patient was admitted to the hospital on February 27, 2023. He was conscious and oriented with a Glasgow Coma Scale score of 14 points and with reactive isocoric pupils, tachypnea, no added sounds on auscultation, rhythmic heart sounds, normal abdomen, and no edema in extremities. No abnormalities at the EKG were reported, and no other symptomatology was associated. He was transferred to the intensive care unit (ICU) from the emergency department due to decompensated heart failure and a suspicion of pulmonary embolism. The patient was awake and oriented and on a high-flow nasal cannula with a fraction of inspired oxygen (FiO_2_) of 50%. The chest computed tomography (CT) scan showed interstitial pneumonia and signs of overload and no signs of pulmonary embolism (Figure 1 and Figure 2).

**Figure 3:**
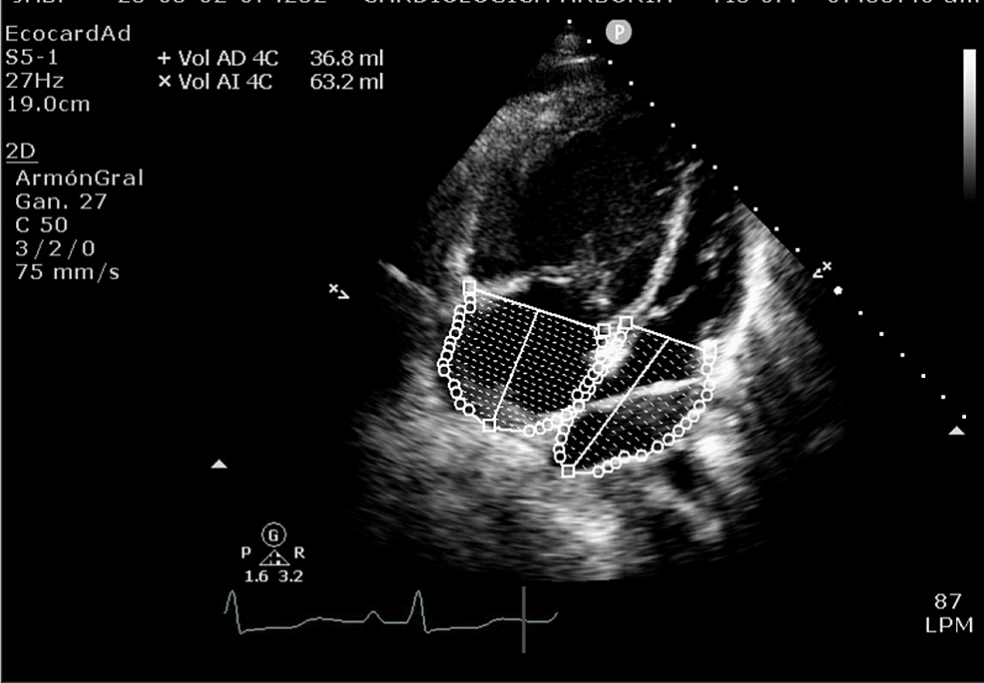
Echocardiogram of four chambers, visualizes left restrictive cor triatriatum The left restrictive cor triatriatum. Situs solitus, atrial and visceral, in levocardia, with concordant atrioventricular and ventriculoarterial connections in the perforated mode. No evidence of intracardiac shunts. Normal pulmonary and systemic venous connections

**Figure 4:**
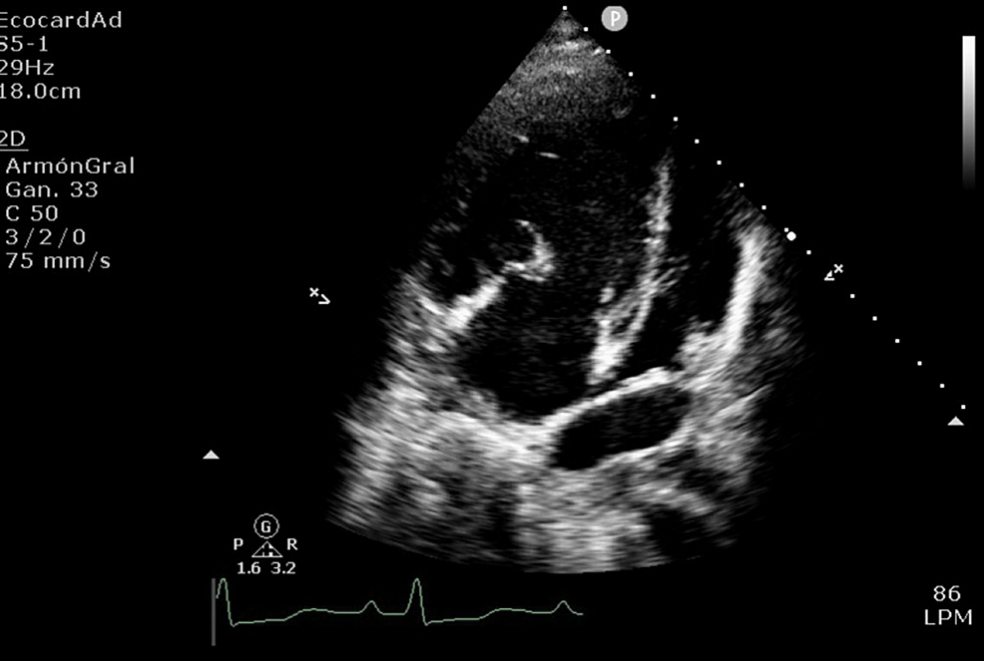
Echocardiogram of cor triatriatum sinister membrane Left atrium: a membrane is observed within the left atrium that divides it into two chambers, a posterior one that receives blood flow from the pulmonary veins and an anterior one in continuity with the mitral valve; both chambers are connected by a small orifice with a diameter of less than 10 mm. The atrial membrane causes severe obstruction of blood flow from the pulmonary veins to the mitral valve. Right atrium: normal dimensions with a slight increase in estimated pressure. A prominent Eustachian valve is observed

A transthoracic echocardiogram, on March 1, 2023, revealed cor triatriatum sinister, pulmonary arterial hypertension with an estimated pulmonary artery systolic pressure (PASP) of 118 mmHg, chronic cor pulmonale, left ventricular morphological changes, and reduced right ventricular function (Figure 3 and Figure 4).

The patient was discharged from the intensive care unit without the need for invasive mechanical ventilation. After three days of treatment with levofloxacin 1 g/day, the management with antibiotic therapy and the request for cardiovascular surgery for the cor triatriatum sinister were made at the internal medicine service. Seven days after the successful surgery at the Dr. Ignacio Chávez National Institute of Cardiology, the patient started getting better; the follow-up care continues at the external medical service at the Institute of Health of Chiapas.

## Discussion

We present a rare case of cor triatriatum sinister (CTS), an exceedingly uncommon congenital anomaly that has been reported only a few times previously [1,2]. In this particular variation, one or more pulmonary veins connect directly to the true left atrium (LA), leading to a significant reduction in the transpulmonary pressure gradient and delaying the onset of symptoms. We propose that the emergence of dyspnea and subsequent syncope in our patient resulted from the progressive calcification of the membrane orifice(s) and concurrent chronic congestion in the right-sided pulmonary circulation. Initially, CTS may be mistaken for dilated coronary sinus or pulmonary veins on echocardiography or even for malignancy or thrombosis. However, the careful examination of the lesion using color Doppler imaging or the use of contrast-enhancing agents during ultrasound can facilitate the diagnosis using echocardiography alone. Transesophageal echocardiography offers superior visualization of the LA and its membrane compared to transthoracic echocardiography, aiding in accurate diagnosis [3,4]. The physiological consequences of cor triatriatum sinister are directly proportional to the size of the opening between the accessory and true atrial chambers. When the foramen is small, the obstruction is significant enough to create a pressure gradient within the atria, mimicking mitral stenosis. Relevant symptoms may also be related to associated cardiac abnormalities [4,5].

In infants and newborns, the manifestations of the disease are secondary to a relatively narrow opening, leading to a subsequent rise in proximal left atrial pressure and pulmonary congestion. Dyspnea ranges from mild forms to more severe presentations such as neonatal respiratory distress, with an increased mortality risk.

Adults with the disease are usually asymptomatic due to the presence of a large foramen without an intra-atrial pressure gradient. The appearance of symptoms occurs secondary to fibrosis and the calcification of the accessory membrane orifice, although the latter is more often obstructive at a younger age before the degenerative changes take place. Symptoms include exertional dyspnea, orthopnea, and hemoptysis. Several cases of pulmonary edema during labor have also been reported in young adult females with initially undiagnosed cor triatriatum sinister. On a systematic review [4-6], approximately 17.5% of patients were asymptomatic at the time of diagnosis. Adults with cor triatriatum sinister most frequently present with exertional dyspnea, orthopnea, and palpitations. Symptoms related to congestive heart failure were present in 26.9% of patients. These late symptoms may be related to the development of supramitral inflow obstruction, mitral regurgitation, pulmonary hypertension, or atrial fibrillation. Ischemic or thromboembolic events are the most typical clinical findings in adults. Syncope was described in a single case report [4].

In our patient, critical care was useful to improve the diagnosis and make a better management and surgical treatment. The surgical outcomes for cor triatriatum sinister are generally favorable in experienced centers, with the majority of patients becoming asymptomatic during follow-up and an overall reported survival rate exceeding 90% at five years [5,6].

In a single-center study of 32 consecutive patients who underwent surgical repair from 1993 to 2020, the overall survival rate was 96.9% at 15 years post repair. Most survivors (96.9%) were in New York Heart Association functional class I at a median follow-up of 74 months. At the latest echocardiography performed at a median of 42 months after repair, no residual lesion was observed except in one patient who had moderate pulmonary hypertension [6,7].

Another study reported a five-year survival rate of 100% in the biventricular group and 82.1% in the univentricular group after cor triatriatum resection [8]. These findings suggest that the surgical repair of cor triatriatum sinister can be performed safely and effectively in experienced centers, with a low risk of recurrence and excellent long-term outcomes for most patients.

There are currently no established protocols for managing the risk of pulmonary embolism in cor triatriatum sinister (CTS), but we believe that anticoagulation therapy should be started very quickly if a patient with CTS experiences an embolic episode, even if they do not have documented atrial fibrillation or a visible atrial blood clot. The treatment for CTS depends on the severity of the patient's symptoms. If an atrial membrane is found incidentally without a pressure gradient, no treatment is necessary. The surgical removal of the atrial membrane may be required for patients with severe obstruction, and this procedure has been shown to provide good short-term and long-term survival rates with a low chance of needing additional surgery in the future.

## Conclusions

The patient was admitted to the intensive care unit for decompensated heart failure and the suspicion of acute pulmonary embolism and was found to have cor triatriatum sinister, pulmonary arterial hypertension, chronic cor pulmonale, and reduced right ventricular function. Despite the severity of his condition, the patient did not require invasive mechanical ventilation and was eventually discharged from the intensive care unit. This case highlights the importance of prompt medical attention for unexplained symptoms, as timely diagnosis and treatment can significantly impact patient outcomes.
